# JAK kinase inhibitors for the treatment of acute lymphoblastic leukemia

**DOI:** 10.1186/s13045-015-0192-7

**Published:** 2015-07-26

**Authors:** Sandrine Degryse, Jan Cools

**Affiliations:** VIB Center for the Biology of the Disease, Leuven, Belgium; KU Leuven Center for Human Genetics, Leuven, Belgium; Herestraat 49 (Box 602), 3000 Leuven, Belgium

**Keywords:** Tyrosine kinase, Cytokine receptor, Oncogenes, Targeted therapy, Kinase inhibitor, Resistance, Mouse model, Cancer, Leukemia

## Abstract

Recent studies of acute lymphoblastic leukemia have identified activating mutations in components of the interleukin-7 receptor complex (IL7R, JAK1, and JAK3). It will be of interest to investigate both JAK1 and JAK3 kinase inhibitors as targeted agents for these leukemias.

## The importance of interleukin-7 receptor signaling for B and T cell development in normal and malignant conditions

Cytokine signaling, orchestrated by various ligands and more than 30 different receptors, plays a critical role during hematopoiesis. Cytokines that bind to receptors containing the common gamma chain (IL2RG) such as IL2, IL4, IL7, IL9, IL15, and IL21 are important for B and T cell development and function [1]. The IL7 receptor has received much of attention because it is a marker for early lymphoid progenitor cells, it is essential for both B and T cell development, and it was recently identified as a dominant oncogene in acute lymphoblastic leukemia (ALL). We will focus here on the IL7 receptor complex as an example of how cytokine receptor signaling can be hijacked by leukemia cells.

**Fig. 1:**
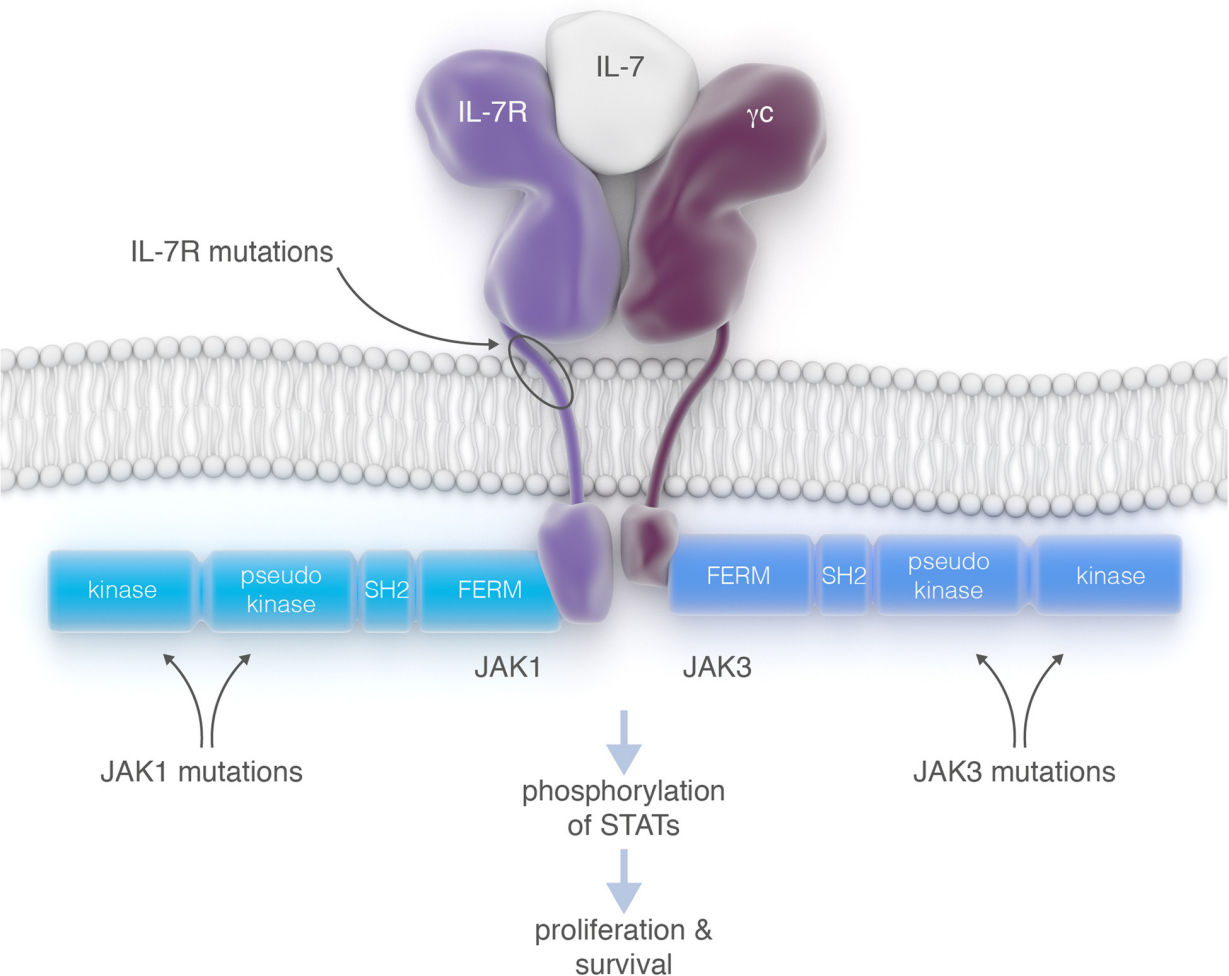
Schematic representation of the interleukin-7 (IL7) receptor signaling complex. *Arrows* indicate locations that are often found to be mutated. Activation of the receptor complex by ligand binding or by mutation in IL7R, JAK1, or JAK3 causes phosphorylation of STAT proteins, which leads to activation of survival and proliferation pathways

The IL7 receptor is a heterodimeric receptor consisting of the IL7 receptor alpha chain (IL7R) and the common gamma chain (IL2RG). These receptor units associate with Janus kinase 1 (JAK1) and JAK3, respectively, and these kinases are both activated upon binding of IL7 to the receptor (Fig. 1). Loss-of-function mutations in IL2RG, IL7R, or JAK3 lead to impaired B and T cell development and have been identified in patients with severe combined immunodeficiency disease, clearly illustrating that this signaling axis is essential for normal lymphocyte development [1]. In contrast, gain-of-function mutations in IL7R, JAK1, or JAK3 lead to ligand-independent activation of IL7 receptor signaling, and have been identified in ALL and in various lymphoma types.

Activating mutations in IL7R are frequently found in T cell ALL (T-ALL) and B cell ALL (B-ALL) [2, 3]. These IL7R activating mutations are located in exon 6 and mostly lead to the incorporation of an unpaired cysteine close to the transmembrane domain of the receptor (Fig. 1). In this way, the mutant IL7R protein can form homodimers by the formation of disulfide bonds resulting in cytokine-independent activation of the downstream signaling pathways. In a minority of ALL cases, the IL7R mutations do not involve the insertion of a cysteine amino acid, and those insertions occur within the transmembrane domain, most likely resulting in ligand-independent activation of heterodimeric receptors.

In addition to mutations in the receptor itself, also mutations in the tyrosine kinase JAK3 are frequent in T-ALL [2, 4, 5], while JAK1 activating mutations occur at a low frequency in T or B ALL (Fig. 1) [2, 6]. Due to the restricted expression pattern of IL7R, IL7R mutations are limited to lymphoid malignancies, while JAK1 and JAK3 mutations could also be expected in myeloid leukemias and even in any type of cancer. Indeed, JAK1 mutations have also been detected in a variety of epithelial tumors, with the highest frequency in hepatocellular carcinoma (http://cancer.sanger.ac.uk).

## Targeted treatment strategies

Over the last decades, combination chemotherapy has been optimized for the treatment of ALL, and childhood ALL can now be cured in more than 80 % of children. Patients, however, suffer from serious short-term and long-term side effects of intensive treatment, and adult ALL patients have a poor outcome. With an increasing understanding of the molecular defects implicated in the pathogenesis of ALL, it is now possible to design patient-specific therapies where treatment is based on the mutational status of the leukemia cells. Since the IL7 receptor complex (JAK1, JAK3, IL7R) is mutated in up to 25 % of the T-ALL cases, this could be one of the new therapeutic targets to be explored.

Protein tyrosine kinases are interesting proteins from a therapeutic perspective, because these enzymes are easy to target with small molecule inhibitors and these proteins are often mutated and constitutively activated in cancer. The ABL inhibitor imatinib has revolutionized treatment of chronic myeloid leukemia, and also in BCR-ABL positive ALL, the combination of ABL kinase inhibitors with chemotherapy has shown promising results. Since the initial successes with imatinib, many other kinase inhibitors have been developed, including a variety of JAK kinase inhibitors (Table 1). While most of these inhibitors are still under development, the JAK1/JAK2-selective inhibitor ruxolitinib is already FDA approved for treatment of patients with myelofibrosis, and the JAK3-selective inhibitor tofacitinib received FDA approval for the treatment of patients with rheumatoid arthritis. These data demonstrate that JAK kinase inhibitors can be administered safely and open new possibilities for the treatment of T-ALL with IL7R, JAK1, or JAK3 mutations. With T-ALL being a rare leukemia, it is very fortunate that numerous JAK inhibitors are already available and could potentially be repurposed for the treatment of T-ALL.

**Table 1:** JAK1 and JAK3-selective inhibitors currently in clinical studies

Name	Selectivity	Patient group	Clinical phase
Baricitinib (LY3009104)	JAK1/2	Rheumatoid arthritis	Phase 3
		Psoriasis	
Ruxolitinib (INCB18424)	JAK1/2	Acute leukemia	Phase 1/2
		Chronic myeloid leukemia (CML)	
		Acute myeloid leukemia (AML)	FDA approved
		Myelofibrosis	
Decernotinib (VX-509)	JAK3	Rheumatoid arthritis	Phase 2/3
Tofacitinib (CP-690550)	JAK3	Rheumatoid arthritis	FDA approved
INCB039110	JAK1	Primary myelofibrosis	Phase 2
		Post-polycythemia vera fibrosis	
		Post-essential thrombocythemia myelofibrosis	
PF-04965842	JAK1	Plaque psoriasis	Phase 2
Filgotinib (GLPG0634)	JAK1	Rheumatoid arthritis	Phase 2
		Crohn’s disease	
INCB047986	JAK1	Rheumatoid arthritis	Phase 2
Momelotinib (CYT387)	JAK1/JAK2	Primary myelofibrosis	Phase 1/2
		Post-polycythemia vera myelofibrosis	Phase 2
		Post-essential thrombocythemia myelofibrosis	
		Polycythemia vera	
		Essential thrombocythemia	Phase 2
GSK2586184	JAK1	Psoriasis	Phase 2
		Systemic lupus erythematosus	
AT9283	JAK2/JAK3	Multiple myeloma	Phase 2
		Acute myeloid leukemia	Phase 1/2
		Acute lymphoblastic leukemia	
		Chronic myeloid leukemia	
		Myelodysplastic syndromes	
		Myelofibrosis	

It has been reported that JAK1 [7], IL7R [3], and JAK3 [8] mutants are sensitive to JAK-selective inhibition. JAK1 is indispensable for IL7R mutants in order to maintain activation of downstream proteins such as STAT5 [3]. Similarly, we reported several lines of evidence that JAK1 is required for the transforming mechanisms of most JAK3 mutants. Thus, although the number of ALL patients with specific mutations in IL7R, JAK1, or JAK3 is low, all these cases together represent about 27 % of T-ALL cases and are all likely to respond to JAK inhibitors [9].

## In vivo models for testing potential compounds

We recently showed that most JAK3 mutations, identified in T-ALL patient samples, caused leukemia in a mouse model [8]. In the development of mouse models expressing JAK3 mutations, we mainly focused on the JAK3 M511I mutation, which is the most common mutation found in T-ALL. Mice receiving bone marrow transplantation with cells expressing the JAK3 M511I mutant developed a lymphoproliferative disease over the first 12 weeks, followed by progression to an acute phase characterized by a rapid increase in white blood cell (WBC) counts. All animals eventually succumbed to the disease within 14 to 28 weeks after receiving the bone marrow transplantation [8]. The disease was characterized by splenomegaly, enlarged thymus, and enlarged lymph nodes. All mice showed an accumulation of CD8 single positive immature T cells in the peripheral blood and hematopoietic tissues. The leukemic cells were transplantable to secondary recipient mice and were characterized by the presence of additional mutations in Notch1, Pten, Kras, and other genes. Mice which received a bone marrow transplant of cells expressing wild-type JAK3 did not develop any disease phenotype [8].

Primary and secondary transplanted mice were subsequently used to test the efficacy of the JAK3-selective inhibitor, tofacitinib (Xeljanz, Pfizer), to treat leukemia progression. Mice treated with tofacitinib showed a decrease in WBC count, while mice receiving placebo treatment had an increase in WBC count during treatment. Pathological analysis of tissues showed a high percentage of apoptotic cells in tofacitinib-treated mice, while placebo-treated mice had very low amount of apoptotic cells. Spleen and thymus weight was significantly lower in tofacitinib-treated mice compared to placebo-treated mice. These data demonstrate that JAK inhibitors such as tofacitinib show activity in mouse leukemia models [8]. However, when treatment was stopped, WBC counts increased, and all animals eventually succumbed to the disease, showing that kinase inhibitors alone cannot lead to complete eradication of the leukemia cells in this mouse model.

In a separate study, Maude and colleagues investigated the efficacy of ruxolitinib for the treatment of early T cell precursor ALL (ETP-ALL) using xenografted human leukemia samples. Injection of ETP-ALL samples in immune deficient NSG mice led to the expansion of the leukemia cells in vivo, which was observed by increasing numbers of human blast cells in the peripheral blood and spleen of the NSG mice over time. Treatment of these animals with ruxolitinib, a JAK1/JAK2 inhibitor, caused a dramatic reduction of peripheral and spleen blasts, even as a single agent. Interestingly, the efficacy of ruxolitinib was observed not only in three samples with JAK1 or JAK3 mutation, but also in two ETP-ALL samples without JAK1, JAK3, or IL7R mutation [10]. These data indicate that JAK inhibitors are promising agents for the treatment of T-ALL, and that clinical trials to test these agents are warranted. JAK1, JAK3, or IL7R mutations predict response to JAK inhibitors, but even T-ALL cases without such mutations could potentially respond to JAK inhibition, most likely due to the presence of other, yet unknown, mechanisms leading to activation of the JAK/STAT pathway.

## What about resistance?

Targeted agents such as tyrosine kinase inhibitors are very active and specific and typically show strong anti-cancer effects, but suffer from the fact that they bind to the target kinase in one specific part, typically the ATP-binding pocket. As a consequence, oncogenic kinases can become resistant to the inhibitor by mutating a single amino acid, a problem that has been observed in nearly all clinical applications of targeted cancer treatment.

It is too early to tell whether JAK inhibitors would show clinical efficacy for the treatment of T-ALL, but in case this is successful, can we also expect resistance and how could we design the best inhibitors? Would it be possible to design a strategy to prevent or limit the development of resistance? The intimate interplay between the JAK1 and JAK3 kinase at the IL7 receptor may offer opportunities for the prevention of resistance development. A recent study showed that cells transformed by a JAK3 mutant could become resistant to a JAK3-selective inhibitor by acquiring another activating mutation in JAK1 [11]. By targeting both JAK1 and JAK3 in JAK3 mutant leukemia cells, it may be very difficult for the leukemia cells to overcome this block by just one mutation. Moreover, our study showed that combination of JAK3-selective and JAK1-selective inhibitors had a clear synergistic effect on the growth of JAK3 mutant cells [8]. In this way, the dose of both drugs could be lowered while still achieving a good inhibition of the signaling pathways.

While combined inhibition of both JAK1 and JAK3 could be beneficial for the treatment of a subset of the T-ALL cases, it is also clear that another group of mutants are very much dependent on JAK1 or JAK3 only. It will be important to further clarify the mechanisms by which the different JAK1, JAK3, and IL7R mutants transform cells. Better insight in the exact signaling pathways that are activated by each of the mutants could help the rational selection of targeted agents to achieve better responses and prevent the development of resistance.

## Abbreviations

ALL: acute lymphoblastic leukemia
B-ALL: B cell acute lymphoblastic leukemia
ETP-ALL: early thymic precursor acute lymphoblastic leukemia
STAT: signal transducer and activator of transcription
T-ALL: T cell acute lymphoblastic leukemia
WBC: white blood cell
